# Troponins in scuba divers with immersion pulmonary edema

**DOI:** 10.1042/BSR20181024

**Published:** 2018-09-28

**Authors:** Marion Marlinge, Pierre Deharo, Fabrice Joulia, Mathieu Coulange, Donato Vairo, Marine Gaudry, Mylene Egensperger, Laura Belkhiri, Myriam Zouggarh, Laurie Bruzzese, Julien Fromonot, Théo Charnay, Camille Petit, Claire Guiol, Giovanna Mottola, Amin Ben Lassoued, Alain Boussuges, Régis Guieu, Pierre Louge

**Affiliations:** 1 Laboratory of Biochemistry, Timone Hospital, Centre for Cardiovascular Disease and Nutrition, Bvd J Moulin, Marseille 13005, France; 2 Department of Cardiology, Timone Hospital, Centre for Cardiovascular Disease and Nutrition, Bvd J Moulin, Marseille 13005, France; 3 Toulon University, Centre for Cardiovascular Disease and Nutrition, Bvd J Moulin, Marseille 13005, France; 4 Department of Hyperbaric Medicine, Sainte Marguerite Hospital, Centre for Cardiovascular Disease and Nutrition, Bvd J Moulin, Marseille 13005, France; 5 Department of Vascular Surgery, Centre for Cardiovascular Disease and Nutrition, Bvd J Moulin, Marseille 13005, France; 6 Diving and Hyperbaric Center, Geneva University Hospital, Centre for Cardiovascular Disease and Nutrition, Bvd J Moulin, Marseille 13005, France

**Keywords:** cardiac failure, immersion pulmonary edema, troponin

## Abstract

Immersion pulmonary edema (IPE) is a serious complication of water immersion during scuba diving. Myocardial ischemia can occur during IPE that worsens outcome. Because myocardial injury impacts the therapeutic management, we aim to evaluate the profile of cardiac markers (creatine phosphokinase (CPK), brain natriuretic peptide (BNP), highly sensitive troponin T (TnT-hs) and ultrasensitive troponin I (TnI-us) of divers with IPE. Twelve male scuba divers admitted for suspected IPE were included. The collection of blood samples was performed at hospital entrance (T0) and 6 h later (T0 + 6 h). Diagnosis was confirmed by echocardiography or computed-tomography scan. Mean ± S.D. BNP (pg/ml) was 348 ± 324 at T0 and 223 ± 177 at T0 + 6 h (*P*<0.01), while mean CPK (international units (IUs)), and mean TnT-hs (pg/ml) increased in the same times 238 ± 200 compared with 545 ± 39, (*P*=0.008) and 128 ± 42 compared with 269 ± 210, (*P*=0.01), respectively; no significant change was observed concerning TnI-us (pg/ml): 110 ± 34 compared with 330 ± 77, *P*=0.12. At T0 + 6 h, three patients had high TnI-us, while six patients had high TnT-hs. Mean CPK was correlated with TnT-hs but not with TnI-us. Coronary angiographies were normal. The increase in TnT during IPE may be secondary to the release of troponin from non-cardiac origin. The measurement of TnI in place of TnT permits in some cases to avoid additional examinations, especially unnecessary invasive investigations.

## Introduction

Immersion pulmonary edema (IPE) is a rare but serious complication of water immersion during snorkeling, apnea, swimming [1], or scuba diving [2]. Cases are often observed amongst professional including military frogmen and triathletes [2–4]. However, the incidence is difficult to determine because this syndrome is often misdiagnosed although sometimes it can be fatal [5], which justifies a rapid diagnosis. Symptomatology includes dyspnea, cough, hemoptysis, blood-tinged sputum, cyanosis, and sometimes fainting. Most patients recover from IPE with appropriate treatment including oxygen, β-mimetic, diuretics, and/or aerosols [6]. The pathophysiology of this syndrome remains elusive but implies hemodynamic and cardiorespiratory disorders leading to the leakage of water into the pulmonary alveolar interstitial spaces [3,7]. In scuba divers, the water immersion associated with hydrostatic pressure in a cold environment leads to environment-induced stress [2,8].

Muscular exercise leads to increase in muscular and cardiac work that contribute to IPE. Risk factors have been reported including hypertension [8,10] and age [9]. Myocardial ischemia has been also reported during IPE that may be associated with worse outcome. Thus, an increase in T troponin has been reported [9] that may be attributed to reversible myocardial ischemia in a Takotsubo context [11]. Because myocardial injury impacts the therapeutic management, cardiac biomarkers could help in screening of patients with IPE. As an example, it was shown that cardiac biomarker profile may help to distinguish IPE from decompression sickness [6]. Thus, the aim of the present study was to evaluate the profile of cardiac biological markers of divers in a context of IPE, and specially the behavior of brain natriuretic peptide (BNP), creatine phosphokinase (CPK), and troponins.

## Methods

### Patients

Scuba divers (12 males, mean age: 51, range: 41–56 years) suspected of IPE admitted at the Department of Hyperbaric Medicine (Sainte-Anne Hospital, Toulon, France) between July 2013 and June 2014 were successively included. Mean dive duration was 21 min (range: 11–24 min), and mean depth was 15 m (range: 5–40 m). Informed consent was obtained from each patient and the study protocol conforms to the ethical guidelines of the 1975 Declaration of Helsinki in *a priori* approval by the institution’s human research committee.

### Biological investigations

Blood samples collection was performed at hospital entrance (T0) and 6 h later (T0 + 6 h). Blood samples were collected using lithium heparinate tubes (plasma) for troponins and CPK, while blood samples were collected using EDTA-K_3_ tubes (plasma) for BNP measurement. All parameters were measured in less than 2 h after blood samples collection. Highly sensitive troponin T (TnT-hs, Elecsys®) and CPK were measured on COBAS-8000 Roche®, while ultrasensitive troponin I (TnI-us) and BNP were measured using ADVIA-Centaur® (Siemens®, Munich Germany). TnI-us was measured using an immunochemical method. This immune-dosage uses one polyclonal antibody and detection was by chemiluminescent signal. The detection threshold was 6 pg/ml (range: 6**–**50 pg/ml; intra-assay variation: **<** 10%; intra-assay range: between 3 and 5%; 99th percentile was 47 pg/ml). TnT-hs was measured using an immunological sandwich method (detection threshold: 5 pg/ml, range: 5–50 ng, intra-assay variation < 10%; intra-assay range: between 2 and 4%; 99th percentile: 14 pg/ml). BNP was measured using a two-site sandwich immunoassay (detection threshold: 2 pg/ml; range 2–2000 pg/ml; intra-assay variation: 10%, inter-assay CV range: between 5 and 10%). CPK was measured by enzymatic dosage. The intra-assay CV was <10% and the inter-assay range was between 2 and 4%. Normal values for CPK and BNP were <192 international units (IUs) and <100 pg/ml, respectively.

### Statistical analysis

Data were expressed as means ± S.D. or median and range. For intra-individual comparisons, because each patient was his own control, the non-parametric Wilcoxon’s test was used. No inter-individual statistical analysis was performed. The Pearson’s coefficient for correlation analysis was used for correlation analysis. *P*-values <0.05 were considered significant. We used Prism software version 5.0a for statistical analysis. The protocol followed the guidelines of the Declaration of Helsinki.

## Results

**Figure 1:**
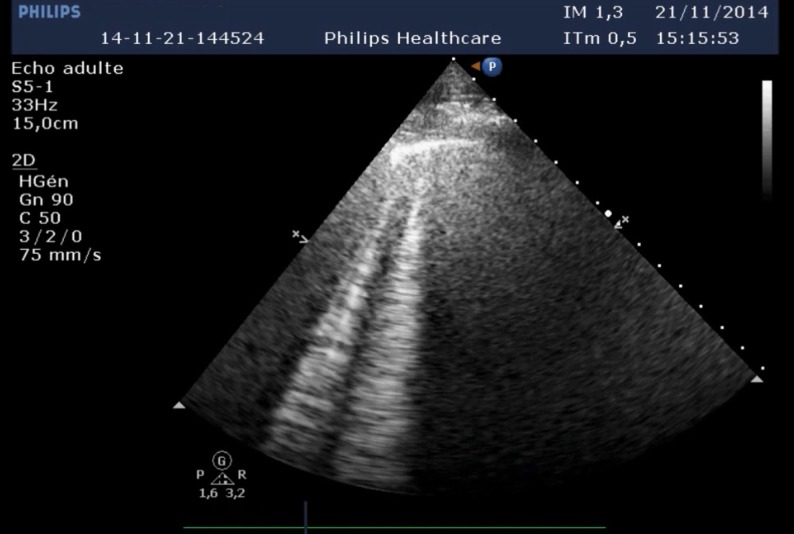
Example of comet pictures visualized by transthoracic echocardiography in a patient hospitalized

Clinical data are given in Table 1. None of the patients had typical chest pain and the ECGs were without ST segment abnormalities. Transthoracic echocardiography revealed no myocardial dyskinesia. IPE diagnosis was confirmed by the presence of interstitial infiltrates on chest computed-tomography scan and/or by the presence of lung comets by chest sonography (see Figure 1).

**Table 1:** Clinical characteristics of patients at the hospital entrance

Patients (12 men)	
Mean age (years), range: 51 (41–56)	
Clinical manifestations	
Anxiety (n=12)	
Dyspnea (*n*=12)	
Cough (*n*=6)	
Blood tinged-sputum (*n*=3)	
Cyanosis (*n*=3)	
Loss of consciousness (*n*=1)	
Systolic blood pressure (mm Hg/mean ± S.D.)	150 ± 18
Saturation (%)	92 ± 8
PO_2_ (*K*Pa)	9210 ± 1860

**Figure 2:**
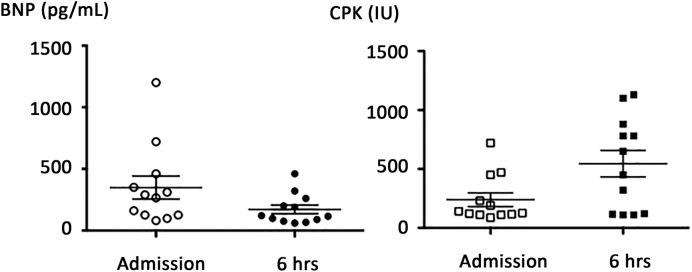
BNP and CPK plasma levels at hospital admission and 6 h later in 12 scuba divers suspected of IPE

**Figure 3:**
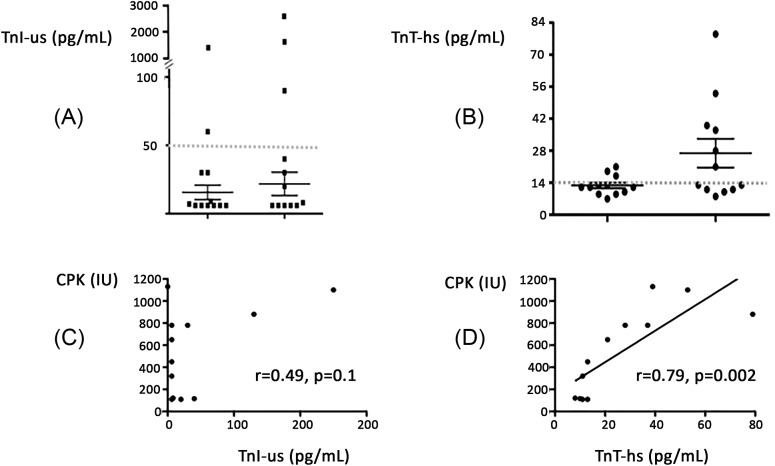
Troponin T (TnT-hs) (A) or Troponin I (TnI-us) (B) at hospital admission and 6 h later in 12 scuba divers suspected of IPE (dotted line: 99th percentile). Correlation curve between highly sensitive (TnT-hs) (C), or highly sensitive troponin I (TnI-us) (D) and CPK evaluated 6 h after hospital admission.

Mean BNP (pg/ml) was 348 ± 324 at T0 and 223 ± 177 at T0 + 6 h (*P*<0.01, see Figure 2), while mean CPK (IU, Figure 2) and mean TnT-hs (pg/ml) increased in the same times 238 ± 200 compared with 545 ± 39 (*P*=0.008) and 128 ± 42 compared with 269 ± 210, (*P*=0.01), respectively; no significant change was observed concerning TnI-us (pg/ml): 110 ± 34 compared with 330 ± 77, *P*=0.12. Regarding TnI-us, two patients (16%) had concentrations over the 99th percentile at T0 and 3 (25%) at T0 + 6 h (Figure 3A); regarding TnT, at T0, three patients (25%) had TnT-hs over the 99th percentile and 6 (50%) at T0 + 6 h (Figure 3B). At time T0 + 6 h, we found no correlation between CPK and TnI-us (see Figure 3C, *r* = 0.49, *P*=0.1), while a correlation was found between CPK and TnT-hs (0.79, *P*=0.002, see Figure 3D). Coronary angiographies were performed in patients with troponin level (TnI-us or TnT-hs) over the 99th percentile, and were normal. All patients were treated with oxygen for 2 and 8 h with β-mimetics, diuretics, and/or aerosols. No complications were observed and patients left hospital after 24 h admission.

## Discussion

The main finding of the present study is the discordance between the number of TnI-us and TnT-hs plasma levels over the 99th percentile in scuba divers with IPE, especially 6 h after hospital admission, and the lack of significant change in TnI-us conversely to TnT-hs.

Multiple reports have examined the behavior of cardiac biomarkers after physical exertion.

Post-exercise release of TnT or TnI has been reported after endurance swimming exercise [1] or triathletes [12,13] mostly during competition. This increase in troponins was attributed to myocardial injury by reactive oxygen species, altered pH, and increase in core temperature [14–16]. The release of troponin may also be the result of an increase in membrane permeability via mechanical stimuli [17]. But such an increase may also be due to the release of TnT from non-cardiac origin, mostly striatal muscles [18]. In this case, the high TnT level is secondary to a cross-reactivity of the antibodies used for dosage, with TnT release from skeletal muscles that occurs during a strong exercise. Indeed, there are clinical and laboratory evidence to show the Roche assay cannot distinguish between cTnT from cardiac muscle and that from diseased skeletal muscle [19]. Whereas injury to myocardium causes the simultaneous release of both cTnI and cTnT into the circulation, injuries to skeletal muscle cause the release of cTnT only, as predicted by their different biological expressions [20].

Our hypothesis is supported by the correlation between CPK and TnT levels. Furthermore, this may explain the higher incidence of TnT elevation compared with TnI during exercise in the multiple reports [17]. In this context, the increase in CPK is likely due to skeletal muscle release.

IPE occurs typically during scuba diving, snorkeling, and swimming. IPE is sometimes associated with myocardial injury or dysfunction [6], mostly in the context of a Takotsubo syndrome triggered by cold immersion, stress, and effort [11]. In these cases, troponin dosage is welcome. But in this context, TnI-us appears more appropriate than TnT-hs.

Finally, high BNP level has been previously reported in IPE [6], sometimes associated with hypertension [8]. Here, we observed a high level in BNP that is consistent with an increased filling pressure.

### Study limitation

The weak size of the patient’s panel is mostly due to the fact that IPE is a rare syndrome. However, the population included was sufficient to perform significant statistical analysis.

## Conclusion

We concluded that increased troponin in context of IPE is mostly not associated with abnormal coronary angiography. Because increased troponin over the 99th percentile, even in the absence of typical chest pain and ST segment abnormality, can lead to coronaro-angiography, the measurement of TnI in place of TnT permit in some cases to avoid additional examinations, especially unnecessary invasive investigations. Finally, based on cTnI only, the existence of myocardial lesions, although mostly present, is probably overestimated.

## Abbreviations

BNP: brain natriuretic peptide
CPK: creatine phosphokinase
cTnT: cardiac troponin T
CV: coefficient of variation
ECG: electrocardiogram
IPE: immersion pulmonary edema
IU: international unit
TnT-hs: highly sensitive troponin T
TnI-us: ultrasensitive troponin I
